# Quantitative flow ratio vs. angiography-only guided PCI in STEMI patients: one-year cardiovascular outcomes

**DOI:** 10.1186/s12872-023-03153-7

**Published:** 2023-03-14

**Authors:** Mindaugas Barauskas, Greta Žiubrytė, Nojus Jodka, Ramūnas Unikas

**Affiliations:** 1 Department of Cardiology, Hospital of Lithuanian University of Health Sciences, Kaunas, Lithuania; 2 Institute of Cardiology, Lithuanian University of Health Sciences, Kaunas, Lithuania

**Keywords:** Quantitative flow ratio, QFR in STEMI, Physiology-guided PCI, QFR-guided PCI, QFR 12-month follow-up, Coronary physiology evaluation in STEMI.

## Abstract

**Background:**

Coronary physiology-guided PCIs are recommended worldwide. However, invasive coronary physiology methods prolong the procedure, create additional risks for the patients, and prolong the fluoroscopy time for an interventional cardiologist. Otherwise, there is a noninvasive coronary physiology evaluation method, QFR, that can be safely used even in STEMI patients.

**Methods:**

A total of 198 patients admitted with STEMI and at least one intermediate (35–75%) diameter stenosis other than the culprit artery between July 2020 and June 2021 were prospectively included in this single-center study. All patients were randomized into one of two groups (1 - QFR-guided PCI; 2 - visual-estimation-only guided PCI). A 12-month follow-up with echocardiography, exercise stress test, and quality of life evaluation was performed in all included patients. For the QOF evaluation, the Seattle Angina Score Questionnaire was chosen. Statistical analysis was performed using the Kolmogorov–Smirnov test, Student’s t-test, Mann–Whitney U test, Pearson’s chi-squared test and Kaplan–Meier estimator.

**Results:**

Ninety-eight (49.5%) patients were randomized to the first group, and 100 (50.5%) patients were included in the second group. Statistically, significantly more patients had a medical history of dyslipidemia (98 vs. 91, p = 0.002) and slightly better left ventricular ejection fraction (42.21 ± 7.88 vs. 39.45 ± 9.62, p = 0.045) in the QFR group. Six fewer patients required non-culprit artery revascularization within the 12-month FU in the QFR group (1.02% vs. 6%, p = 0.047). Survival analysis proved that patients in the Angio group had a more than 6-fold greater risk for death within a 12-month period after MI (OR 6.23, 95% CI 2.20-17.87, p = 0.006), with the highest mortality risk within the first two months after initial treatment.

**Conclusion:**

Using QFR in non-culprit lesions in patients with ST-elevation myocardial infarction reduces mortality and revascularization at the 12-month follow-up and improves the quality of life of the patient.

**Trial registration:**

The study was approved by the Regional Bioethical Committee and conducted under the principles of the Helsinki Declaration and local laws and regulations.

**Supplementary Information:**

The online version contains supplementary material available at 10.1186/s12872-023-03153-7.

## Background

When treating patients who have suffered an ST-segment elevation myocardial infarction (STEMI), the initial percutaneous coronary intervention (PCI) should be performed as quickly as possible, and it is recommended that only infarct-related artery (IRA) PCI be performed [1]. On the other hand, more than half of patients diagnosed with acute myocardial infarction have multivessel disease, which is defined as a diameter stenosis of at least two coronary arteries that is greater than or equal to 50% [2]. As a result, the majority of patients need staged PCI in arteries that were not the culprit. In spite of the fact that it is advised that a second procedure be performed as soon as it is feasible to do so, the urgency of staged PCI is ultimately up to the discretion of the attending physician and is primarily determined by the degree of the lesion. At this time, methods of physiological evaluation are used to assist in determining the significance of lesions.

Despite all improvements, fractional flow reserve (FFR), an invasive hyperemic hemodynamic physiology evaluation method, remains the gold standard for physiological evaluation [3]. However, this method requires extra time for pressure wire manipulation and adenosine to induce hyperemia. All of these factors prolong the procedure time [4] and may trigger complications and stress for patients [5]. Furthermore, the FFR is a highly disorderable method, and its values may be underestimated in patients who consumed caffeine within 24 h, despite requiring a higher dose of adenosine [6]. The use of FFR in patients with ACS remains limited, as during the acute phase and the period up to 6 months, microcirculation dysfunction can determine the reaction of pharmacological vasodilatation [7] and can be present throughout the entire myocardium [4, 8, 9]. These changes may misrepresent the FFR values, increasing them [10, 11]. Ntalianis et al. were the first researchers to confirm FFR compatibility on non-culprit vessels in ACS [12]. The results were similar in both groups [12]. However, the average time between FFR measurements was 35 4 days, which could have an impact on not fully recovered microcirculation, resulting in incorrect FFR values in both [4, 12]. Moreover, Van Belle et al. showed that FFR in the ACS reclassified 38% of revascularization strategies and 39% of strategies in the non-ACS [13]. The FAMOUS-NSTEMI clinical trial demonstrated that FFR should be used to confirm treatment strategy in patients with NSTEMI [14]. The study showed a significantly reduced interventional treatment strategy in the FFR group compared to the initial decision [14]. Additionally, Hakeem et al. demonstrated that an FFR value < 0.84 during ACS was the cut-off for stenoses, which would significantly reduce major adverse cardiovascular events (MACEs) in the future [15]. They concluded that FFR measured between 0.8 and 0.85 in ACS patients should be confirmed before planning treatment [15].

Furthermore, compared to angiography-guided PCI, FFR-guided PCI significantly reduces reinfarction and mortality rates [16]. FFR-guided PCI can also be cost-effective up to 21% at one year and up to 22% at three years compared to IRA-PCI alone in STEMI patients [17].

The instantaneous wave-free ratio (iFR) is an additional adenosine-free physiology evaluation method that has been introduced in clinical practice because adenosine-caused hyperaemia is very uncomfortable and creates an additional risk for patients. When compared to FFR, iFR is responsible for 27.7% fewer adverse procedural reactions [18].

Two extensive clinical studies compared iFR- and FFR-based PCI in patients with stable coronary artery disease or non-STEMI with a 1-year follow-up [18, 19]. The results were similar and without significant differences [18, 19]. Hoeven et al. compared iFR values in STEMI patients 1 month later. The trial found no significant changes in iFR values during this time period, but it did find significant changes in FFR values in relation to microcirculation dysfunction and hyperaemia [20]. Another trial, however, discovered promising results between iFR classification agreement and time after STEMI [21]. The agreement between acute and within 5 days follow-up iFR classification was 89%, but only 70% between acute and > 16 days follow-up [21]. The physiological changes during STEMI may impact the classification disagreement in the more extended follow-up period [21]. In STEMI patients, it was discovered that iFR could overestimate the severity of stenoses while FFR could underestimate it in the acute and subacute phases [22]. Theoretically, the iFR could be used in ACS patients [23], but it still prolongs procedure time, requires a pressure wire, and makes the procedure costly.

Since physiology evaluation is underused worldwide, it was believed that the wireless physiology evaluation method may become a game changer. Even though it can be used offline, it is suitable for physiological evaluation in hemodynamically unstable patients with cardiogenic shock, in contrast to wire-based methods, which significantly extend the procedure time [12, 20, 21, 24]. One of the options is a novel noninvasive physiological evaluation method known as the quantitative flow ratio (QFR). The index strongly correlates with other methods, especially FFR [25–27]. Retrospective QFR analysis in STEMI patients revealed that if revascularization was performed on a QFR 0.8 rather than angiography-based PCI, 62.9% could avoid the primary endpoints in 5 years [28]. Furthermore, QFR-based non-infarct-related artery PCI avoids 10.5% of the primary endpoints at 12 months compared to IRA revascularization alone [29]. Lauri et al. recommend using QFR as the first-choice physiological assessment method, switching to FFR only in debatable cases [30].

Despite all novelties, physiology-guided PCIs, especially in ACS patients, remain underused worldwide. Therefore, this study was designed to compare the cardiovascular outcomes of visually estimated only-guided PCI versus noninvasive physiology assessment-guided PCI. As a result, the purpose of the study was to investigate the differences between the non-culprit lesion significance evaluation methods in terms of the quality of life (QOL), the rate of rehospitalization, and the rate of revascularization within the first 12 months following the initial treatment.

## Methods

We prospectively enrolled 198 multivessel diseased STEMI patients who were admitted to our center from July 2020 to June 2021 and had a non-culprit stenosis (35–75%).

After giving their written consent, all included patients were randomized into one of two groups: (1) QFR-guided PCI (named QFR) and (2) visual estimation-only guided PCI (named Angio). Patient data, medical history, and medical treatment were collected from their medical records.

All coronary artery angiographies (CAGs) were performed in compliance with the recommendation for QFR analysis as described in previous publications [31].

Visual estimation was performed by a CAG performing doctor and discussed at the interventional cardiologists’ meeting. If any additional physiological evaluation was needed upon meeting the judgment, the patient was excluded from the study and treated following European Society of Cardiology (ESC) guidance.

The quantitative flow ratio is an innovative method to evaluate the hemodynamic significance of coronary stenoses. The evaluation is based on two different angiographic projections. Special software was used to calculate the pressure differences between pre-stenosis and post-stenosis. In this study, stenoses were observed as hemodynamically significant when the QFR was less than 0,8. For those who were randomized to the QFR group, QFR analyses were performed using the same software - Medis Medical Imaging, Medis QFR® 20.0. All of the QFR analyses were performed offline twice by an experienced and internationally certified QFR observer and averaged. If those two measurements were not matching (the difference between the two measurements was > 0.02), the third measurement was performed, and the three were then averaged. None of the patients were unsuitable for QFR analyses; therefore, none of them were excluded from this group.

For the QOL evaluation, the Seattle Angina Score Questionnaire (SASQ) was chosen (Additional File 1). According to SASQ, the physical limitation and angina frequency were classified as minimal (scores 75–100), mild (50–74), moderate (25–49), and severe (0–24).

A 12-month follow-up was performed in all included patients as a phone call if there were any adverse events or complaints within this period. If the patient complained of any new or exacerbated cardiac symptoms, an in-person meeting and additional examination were scheduled.

Patients who could not be reached by phone were mailed a letter addressing them or their relatives as provided on a written consent form. If any response within one month was obtained, they were checked on our hospital’s digital system for adverse events or death.

The primary outcome involved in this study was mortality. Secondary outcomes: rehospitalization for culprit artery and non-culprit artery revascularization within the 12-month follow-up; and physical activity limitations (according to the SASQ) within the 12-month follow-up.

Statistical analysis was performed using SPSS 28.0 software. The Kolmogorov-Smirnov test was used to test quantitative parameters; if they were normally distributed, differences between two groups were evaluated using the Student’s t-test and expressed as the mean with standard deviation (SD); otherwise, they were evaluated using the Mann-Whitney U test and expressed as the median with interquartile range (IQR). Differences between categorical parameters were tested using *Pearson’s chi-squared test*. Survival analysis was performed using the *Kaplan–Meier estimator* and expressed as a Kaplan-Meier survival curve. The chosen level of significance was p < 0.05.

The study was approved by the Regional Bioethical Committee and was done according to the principles of the Helsinki Declaration and local laws and rules.

## Results

Of all, 98 (49.5%) patients were randomized to the QFR-guided PCI group, and 100 (50.5%) patients were included in the angiography-guided PCI group.

**Fig. 1:**
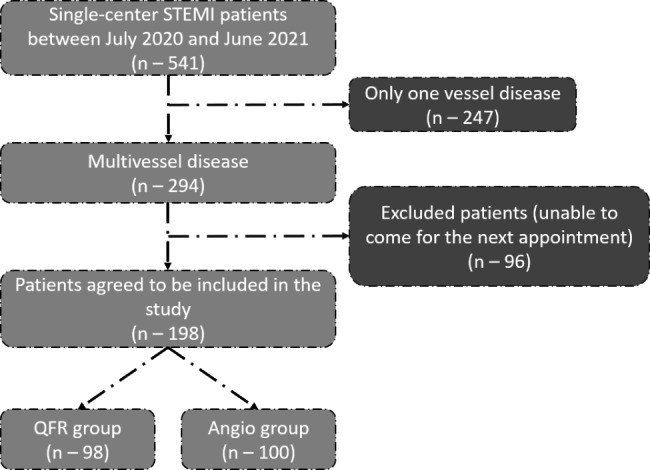
Study flowchart (STEMI - ST elevation myocardial infarction; QFR - quantitative flow ratio)

Significantly more patients had a medical history of dyslipidemia and slightly better left ventricular ejection fraction (LVEF) (Table 1; Fig. 1) in the QFR group. PCI in anamnesis was more common in the Angio group (Table 1). There were no other differences between the two groups (Table 1).

**Table 1:** Patient demographic, physical and medical parameters at admission and during the hospital stay

Parameter	All study population, N = 198	QFR group, N = 98	Angio group, N = 100	p-value
**Age** ***(SD), years***	65,0 (10,60)	64,48 (10,50)	66,46 (10,58)	p = 0.188
**Male**, ***n (%), patients***	140 (70,7)	67 (68,4)	73 (73,0)	p = 0.474
**Female**, ***n (%), patients***	58 (29,3)	31 (31,6)	27 (27,0)	p = 0.474
**Conscious on admission**, ***n (%), patients***	194 (98,0)	95 (96,9)	99 (99,0)	p = 0.303
** h median [IQR]**, ***bpm***	-	72.5 [61.0-85.3]	76.5 [65.3–86.0]	p = 0.189
**Systolic BP median [IQR]**, ***mmHg***	-	80.0[72.3–90.0]	80.0[72.0–90.0]	p = 0.825
**Inferior MI**, ***n (%), number of patients***	104 (52,5)	51 (52,0)	53 (53,0)	p = 0.404
**Anterior MI**, ***n (%), number of patients***	79 (39,9)	37 (37,8)	42 (42,0)	p = 0.404
**Other localization MI**, ***n (%), number of patients***	15 (7,6)	10 (10,2)	5 (5,0)	p = 0.404
**Culprit artery**				
**RCA**, ***n (%), number of patients***	110 (55,6)	58 (59,2)	52 (52,0)	p = 0.625
**LAD**, ***n (%), number of patients***	65 (32,8)	28 (28,6)	37 (37,0)	p = 0.625
**LCX**, ***n (%), number of patients***	18 (9,1)	9 (9,2)	9 (9,0)	p = 0.625
**LM**, ***n (%), number of patients***	5 (2,5)	3 (3,1)	2 (2,0)	p = 0.625
**Serum potassium level**, ***mmol/L (SD)***	3.97 (0.50)	3.98 (0.44)	3.96 (0.55)	p = 0.446
**Serum magnesium level**, ***mmol/L (SD)***	0.83 (0.15)	0.83 (0.15)	0.82 (0.14)	p = 0.446
**AH**, ***n (%), number of patients***	179 (90,4)	91 (92,9)	88 (88,0)	p = 0.246
**DM**, ***n (%), number of patients***	43 (21,7)	17 (17,3)	26 (26,0)	p = 0.140
**Dyslipidaemia**, ***n (%), number of patients***	189 (95,5)	98 (100)	91 (91,0)	**p = 0.002**
**Active smoking**, ***n (%), number of patients***	68 (34,3)	33 (33,7)	35 (35,0)	p = 0.844
**History of MI**, ***n (%), number of patients***	16 (8,1)	6 (6,1)	10 (10,0)	p = 0.317
**History of PCI**, ***n (%), number of patients***	31 (15,7)	6 (6,1)	25 (25,0)	**p < 0.001**
**LVEF**, ***% (SD), percent***	40.82 (8.88)	42.21 (7.88)	39.45 (9.62)	**p = 0.045**
**In-hospital mortality**, ***n (%), number of patients***	6 (3,0)	0 (0)	6 (6,0)	**p = 0.046**

AH – arterial hypertension; BP – blood pressure; DM – diabetes mellitus; HR – heart rate; LAD – left anterior descending artery; LCX – left circumflex artery; LM – left main artery; LVEF – left ventricular ejection fraction; MI – myocardial infarction; PCI – percutaneous coronary intervention; RCA – right coronary artery

During the 12-month follow-up (FU) period, 6 (6%) patients required additional non-culprit MI artery revascularization, which was decided not to treat according to visual estimation in the Angio group (Table 2). At the 12-month follow-up, the majority of the patients continued dual antiplatelet therapy (DAPT) with aspirin and ticagrelor, 91 (92.86%) vs. 84 (84%) in the QFR and Angio groups, respectively. The other 6 (6.12%) patients in the QFR group and 15 (15%) patients in the Angio group used DAPT with aspirin and clopidogrel. Overall, only 1 patient discontinued DAPT while remaining on aspirin only in the QFR group. All patients were on an adequate dose of other post-MI medical treatments, including beta-adrenoreceptor blockers (BABs), angiotensin converting enzyme inhibitors (ACEis), and mineralocorticoid receptor antagonists (MRAs), if needed in both groups at the 12-month FU check-up. Of all, 32 (32.65%) patients in the QFR group and 52 (52%) patients in the Angio group were on oral diuretics (p = 0.006). Patients in the Angio group had a 2.2 times greater risk for the need for oral diuretics for the extended period after MI and visual estimation only of the non-culprit artery (OR 2.23, 95% CI 1.26–3.98). Additionally, patients in the QFR group had an almost 4% better LVEF increase within 6 months after MI compared to visual estimation-only guided PCI. All summarized patient 12-month follow-up data are given in Table 2.

**Table 2:** Follow-up data of the study population

Parameter		All study population, N = 198	QFR group, N = 98	Angio group, N = 100	p-value
**LVEF (6-month FU)**, ***% (SD), percent***		42.63 (10.58)	42.88 (11.78)	42.36 (9.16)	p = 0.213
**Delta-LVEF (6-month FU)**, ***% (SD), percent***		1.96 (4.73)	3.97 (4.30)	-0.06 (4.29)	**p = 0.041**
**Rehospitalization within 12-month FU**, ***n (%), number of patients***		15 (7,6)	5 (5,1)	10 (10,0)	p = 0.153
**Culprit-artery revascularization within 12-month FU**, ***n (%), number of patients***		14 (7,1)	4 (4,1)	10 (10,0)	p = 0.081
**Non-culprit artery revascularization within 12-month FU**, ***n (%), number of patients***		7 (3,5)	1 (1,0)	6 (6,0)	**p = 0.047**
**Class of physical limitations according to SASQ**, ***n (%), number of patients***					
	**Minimal**	84 (42,4)	46 (46,9)	38 (38,0)	p = 0.274
	**Mild**	94 (47,5)	49 (50,0)	45 (45,0)	p = 0.274
	**Moderate**	8 (4,0)	2 (2,0)	6 (6,0)	p = 0.274

FU – follow up; LVEF – left ventricular ejection fraction; SASQ – Seattle angina score questionnaire

**Fig. 2:**
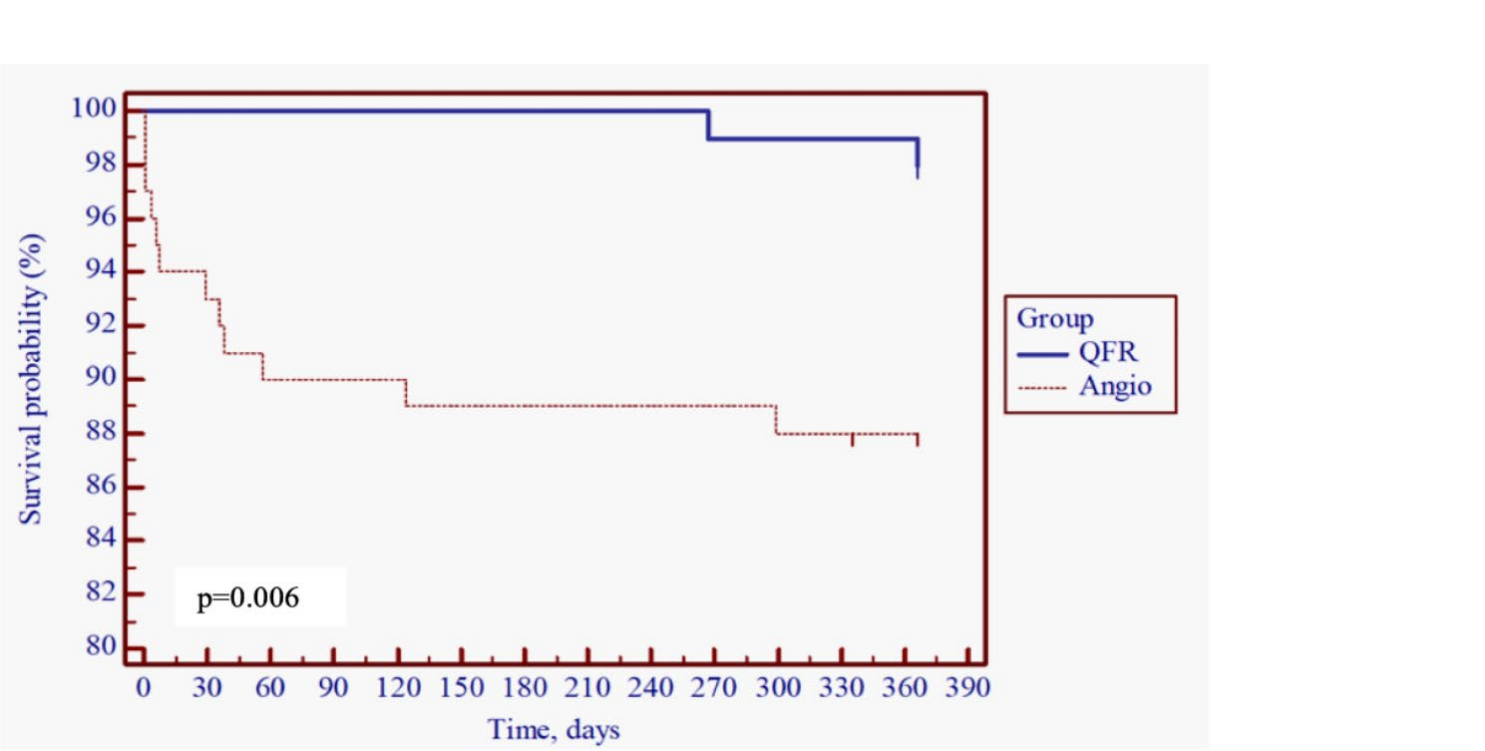
A 12-month survival in the QFR and Angio groups

Within the 12-month period, 14 (7.07%) patients died, 2 (2.04%) in the QFR group and the remaining 12 (12%) in the Angio group. Survival analysis proved that patients in the Angio group had a more than 6-fold greater risk for death within the 12-month period after MI (OR 6.23, 95% CI 2.20-17.87, p = 0.006) (Fig. 2), with the highest mortality risk within the first two months after initial treatment.

## Discussion

In the present study, we compared QFR-guided versus angiography-based PCI on non-culprit lesions over a 12-month follow-up period. The main difference between these groups was the method based on which further clinical decisions were made regarding whether staged PCI in non-culprit arteries was necessary. Otherwise, treatment was identical in both groups. The results showed that patients whose non-culprit arteries were judged according to the QFR values (interventional treatment performed in non-culprit lesions with a QFR ≤ 0.8) had significantly better outcomes and six times less non-culprit lesion revascularization during the follow-up period. Additionally, in the one-year period, the QFR-guided group had twice as many rehospitalizations, and their physical limitations were better (Table 2). As interventional cardiologists acknowledge, physiology-guided PCI is recommended over conventional PCI [32]. This study proved that QFR might be used as a method of choice to increase physiological guidance even in STEMI patients.

Most patients with STEMI are unstable and more fragile than those with NSTEMI. For this reason, PCI for STEMI patients should be as fast as possible and limited to hemodynamically significant stenosis, especially during the index procedure. Invasive coronary physiology methods, such as iFR and FFR, require additional time and manipulations with a pressure wire. Otherwise, QFR is a noninvasive coronary physiology method that can be performed offline, not only by the interventional cardiologist but also by a specialist who is qualified to work with the software. This fact allows interventional cardiologists to perform treatment procedures on culprit lesions while one team member evaluates QFR on non-culprit stenosis. QFR makes the procedure smoother and more accurate, and our study demonstrates that it is likely to be used in STEMI patients.

During the 12-month follow-up period, only five patients in the QFR group were rehospitalized, while twice as many in the Angio group. This shows that this noninvasive coronary physiology method can decrease the rehospitalization rate. A reduced rate of rehospitalizations is one of the main pros of using physiology-guided PCI [33]. Rehospitalizations were related to culprit or non-culprit lesions. In the QFR group, six times fewer patients were hospitalized 12 months after STEMI for non-culprit PCI than in the Angio group. In addition, only one patient required PCI when QFR showed no significant stenosis on the non-culprit artery.

Similar studies to the factors of the follow-up period involved only major adverse cardiovascular events (MACEs) or physiology-guided coronary lesion revascularizations [34, 35]. However, in this study, all patients were followed up for 12 months, and the Seattle Angina Questionnaire was used to objectively identify physical limitations. This criterion is fundamental when investigating coronary physiology methods because physiology-based PCI’s most crucial part is finding hemodynamically significant stenosis and not treating stenosis, which is not substantial. The study results showed that three times fewer patients in the QFR group had moderate physical limitations than those in the Angio group (Table 2). Nevertheless, the patients in the QFR group more often had mild or minimal symptoms of physical activity limitations, according to the Seattle Angina Score Questionnaire. Additionally, all patients in this study underwent an exercise stress test and echocardiography in the following period. All participants in the QFR group had nonpathological exercise stress test results and greater LVEF recovery within 6 months of MI than in the Angio group (Table 2).

The mortality rate is one of the essential facts of the following period. FFR demonstrated this factor’s efficiency and showed us that it is possible to reduce mortality rates using coronary physiology evaluation methods [36]. In this study, the patients in the Angio group died six times more often than patients in the QFR group. Additionally, the results showed that the majority of these patients in the Angio group died during the first two months after STEMI. Therefore, as an FFR, the QFR is efficient in reducing the mortality rate and should be used for all patients with non-culprit artery stenosis.

## Conclusion

The QFR is a noninvasive coronary physiology evaluation method that is accurate for STEMI patients and can be performed by any qualified team member. The use of QFR for patients with ST-elevation myocardial infarction significantly reduced the mortality rate and revascularization at the 12-month follow-up. The patients who underwent PCI guided by the QFR had lower physical activity limitations during their daily activities.

## Electronic supplementary material

Below is the link to the electronic supplementary material.

Additional file: The Seattle Angina Questionnaire

## Data Availability

The datasets generated and analysed during the current study are not publicly available due to patients’ privacy but are available from the corresponding author on reasonable request.
