# Correction: Quantitative flow ratio vs. angiography-only guided PCI in STEMI patients: one-year cardiovascular outcomes

**DOI:** 10.1186/s12872-023-03189-9

**Published:** 2023-03-30

**Authors:** Mindaugas Barauskas, Greta Žiubrytė, Nojus Jodka, Ramūnas Unikas

**Affiliations:** 1 Department of Cardiology, Lithuanian University of Health Sciences, Kaunas, Lithuania; 2 Institute of Cardiology, Lithuanian University of Health Sciences, Kaunas, Lithuania

**Correction: BMC Cardiovascular Disorders 23, 136 (2023)**

**https://doi.org/10.1186/s12872-023-03153-7**

Following publication of the original article [1], in this article “hospital of” has been removed in the first affiliation and the affiliation will read as follows:

Mindaugas Barauskas^1*^, Greta Žiubrytė^1,2^, Nojus Jodka^1^ and Ramūnas Unikas^1^

^1^Department of Cardiology, Lithuanian University of Health Sciences, Kaunas, Lithuania

^2^Institute of Cardiology, Lithuanian University of Health Sciences, Kaunas, Lithuania

The original article has been corrected.
