# Supplementary material for: Quantitative flow ratio vs. angiography-only guided PCI in STEMI patients: one-year cardiovascular outcomes
Source: BMC Cardiovasc Disord. 2023 Mar 14;23:136. doi: 10.1186/s12872-023-03153-7 (PMC10015917; doi:10.1186/s12872-023-03153-7)
Supplement: Supplementary file 1 — Additional file: The Seattle Angina Questionnaire [file 12872_2023_3153_MOESM1_ESM.docx]

1. The Seattle Angina Questionnaire

1. The following is a list of activities that people often do during the week. Although for some people with several medical problems it is difficult to determine what it is that limits them, please go over the activities listed below and indicate how much limitations you have had **due to chest pain, chest tightness, or angina over the past 4 weeks**.

| **Activity** | **Severely Limited** | **Moderately Limited** | **Somewhat Limited** | **A Little Limited** | **Not Limited** | **Limited, or did not do for other reasons** |
| --- | --- | --- | --- | --- | --- | --- |
| **Dressing yourself** |  |  |  |  |  |  |
| **Walking indoors on level ground** |  |  |  |  |  |  |
| **Showering** |  |  |  |  |  |  |
| **Climbing a hill or a flight of stairs without stopping** |  |  |  |  |  |  |
| **Gardening, vacuuming, or carrying groceries** |  |  |  |  |  |  |
| **Walking more than a block at a brisk pace** |  |  |  |  |  |  |
| **Running or jogging** |  |  |  |  |  |  |
| **Lifting or moving heavy objects (20-30 kg)** |  |  |  |  |  |  |
| **Participating in strenuous sports (e.g. swimming, tennis)** |  |  |  |  |  |  |

2. **Compared with 4 weeks ago**, how often do you have **chest pain, chest tightness, or angina** when doing your **most strenuous** level of activity?

| **Much more often** | **Slightly more often** | **About the same** | **Slightly less often** | **Much less often** |
| --- | --- | --- | --- | --- |
|  |  |  |  |  |

3. **Over the past 4 weeks**, on average, how many time have you had **chest pain, chest tightness, or angina**?

| **4 or more times per day** | **1-3 times per day** | **3 or more times per week but not every day** | **1-2 times per week** | **Less than once a week** | **None over the past 4 weeks** |
| --- | --- | --- | --- | --- | --- |
|  |  |  |  |  |  |

4. **Over the past 4 weeks**, on average, how many times have you had to take **nitroglycerin** **tablets** for your **chest pain, chest tightness, or angina**?

| **4 or more times per day** | **1-3 times per day** | **3 or more times per week but not every day** | **1-2 times per week** | **Less than once a week** | **None over the past 4 weeks** |
| --- | --- | --- | --- | --- | --- |
|  |  |  |  |  |  |

5. How bothersome is it for you to take your pills **for** **chest pain, chest tightness, or angina** as prescribed?

| **Very bothersome** | **Moderately bothersome** | **Somewhat bothersome** | **A little bothersome** | **Not bothersome at all** | **My doctor has not prescribed pills** |
| --- | --- | --- | --- | --- | --- |
|  |  |  |  |  |  |

6. How satisfied are you that everything possible is being done to treat your **chest pain, chest tightness, or angina**?

| **Not satisfied at all** | **Mostly dissatisfied** | **Somewhat satisfied** | **Mostly satisfied** | **Highly satisfied** |
| --- | --- | --- | --- | --- |
|  |  |  |  |  |

7. How satisfied are you with the explanations your doctor has given you about your **chest pain, chest tightness, or angina**?

| **Not satisfied at all** | **Mostly dissatisfied** | **Somewhat satisfied** | **Mostly satisfied** | **Highly satisfied** |
| --- | --- | --- | --- | --- |
|  |  |  |  |  |

8. Overall, how satisfied are you with the current treatment of your **chest pain, chest tightness, or angina**?

| **Not satisfied at all** | **Mostly dissatisfied** | **Somewhat satisfied** | **Mostly satisfied** | **Highly satisfied** |
| --- | --- | --- | --- | --- |
|  |  |  |  |  |

9. Over the past 4 weeks, how much has your **chest pain, chest tightness, or angina** interfered with your enjoyment of life?

| **It has severely limited my enjoyment of life** | **It has moderately limited my enjoyment of life** | **It has slightly limited my enjoyment of life** | **It has barely limited my enjoyment of life** | **It has not limited my enjoyment of life** |
| --- | --- | --- | --- | --- |
|  |  |  |  |  |

10. If you had to spend the rest of your life with your **chest pain, chest tightness, or angina** the way it is right now, how would you feel about this?

| **Not satisfied at all** | **Mostly dissatisfied** | **Somewhat satisfied** | **Mostly satisfied** | **Highly satisfied** |
| --- | --- | --- | --- | --- |
|  |  |  |  |  |

11. How often do you worry that you may have a heart attack or die suddenly?

| **I can’t stop worrying about it** | **I often think or worry about it** | **I occasionally worry about it** | **I rarely think or worry about it** | **I never think or worry about it** |
| --- | --- | --- | --- | --- |
|  |  |  |  |  |
